# Exploring the Effects of Acute Digital Sports Dance Intervention on Children’s Gross Motor Development, Executive Function, and Muscle Coordination Using Electromyography Sensors: A Randomized Repeated-Measures Study

**DOI:** 10.3390/s25195962

**Published:** 2025-09-25

**Authors:** Jiao He, Junya Zhao, Haojie Li, Jiangang Chen, Ying Qin

**Affiliations:** 1 School of Art, Wuhan Sports University, Wuhan 430079, China; 2008020@whsu.edu.com; 2 School of Physical Education, Wuhan Sports University, Wuhan 430079, China; zhao77whsu@163.com; 3 School of Exercise and Health, Shanghai University of Sport, Shanghai 200438, China; 202121070037@mail.bnu.edu.cn; 4 College of P.E. and Sprots, Beijing Normal University, Beijing 100875, China; 202131070010@mail.bnu.edu.cn; 5 Graduate School, Wuhan Sports University, Wuhan 430079, China

**Keywords:** EMG sensors, gross motor development, executive function, muscle coordination, electromyography

## Abstract

**Objective:** This paper examines how rhythm-enhanced digital dance affects children’s motor abilities, cognitive performance, and neuromuscular synchronization. **Methods:** In a randomized repeated-measures study, 38 children (7–12 years) underwent three conditions: groove music-accompanied dance (GODA), conventional music dance (CODA), and non-musical physical activity (CON). Assessments of gross motor skills (using TGMD-3), executive function (using BRIEF and Stroop Test), and muscle coordination (using sEMG) were conducted. **Results:** Gross motor skills: GODA showed significantly higher TGMD scores in locomotor (*p* = 0.03) and ball skills (*p* = 0.02) compared to both CODA and CON (*p* < 0.001). Executive function: Inhibition and shifting dimensions showed significant post-intervention condition differences (*p* < 0.05). Muscle coordination: GODA exhibited greater β- and γ-band COH areas in the standing long jump compared to both CODA (*p* = 0.02) and CON (*p* < 0.001), and increased γ-band COH areas in single-leg balance compared to CODA (*p* = 0.02) and CON (*p* < 0.001). **Conclusions:** Combining rhythmic auditory stimulation with movement training offers a promising approach for integrated motor-cognitive development in children.

## 1. Introduction

School-aged children today face two major challenges: over 60% exhibit sedentary behaviors [1], while digital entertainment further reduces physical activity time. As a result, 34% of children worldwide fail to meet gross motor development milestones [2]. Poor motor skills not only delay fundamental abilities such as running and jumping, but may also impede the development of executive functions [3]. For instance, children with motor coordination difficulties often perform worse on cognitive tasks like the Stroop Test [4]. Muscle coordination, which is essential for efficient movement, is frequently underdeveloped in inactive children, increasing their risk of fatigue and injury [5].

Sports dance, with its structured yet creative movements, offers a potential solution. By integrating spatial awareness, rhythm, and social interaction, it engages multiple brain regions [6]. A previous study showed that children who participated in sports dance three times per week for six months improved their Test of Gross Motor Development-Third Edition (TGMD-3) scores by 27% compared to those in traditional physical education (PE) classes [7]. Movements such as Latin dance steps require rapid sensory–motor integration, which may enhance proprioception [8].

Music plays a key role in this process. Rhythms at 120–140 beats per minute (BPM) have been shown to improve movement synchronization in children [9], while moderately irregular tempos (20–30% syncopation) can facilitate motor learning [10]. Groove music—characterized by rhythmic accentuations such as prominent bass lines and syncopation—has been found to enhance motor synchronization and engagement in children [11]. Moreover, the rhythmic pulse of groove music can stimulate dopamine release [12] and reduce motor preparation time by 32% through enhanced brain connectivity [13]. Children demonstrate stronger responses to such rhythms than adults [14,15].

The dual-task nature of sports dance (e.g., moving while maintaining rhythm) may also benefit working memory [16,17]. Electromyography (EMG) studies indicate that skilled dancers use 15–20% less muscle co-activation than novices [18], suggesting that movement efficiency may free cognitive resources and improve cerebellar–prefrontal communication [19].

In this study, we use surface EMG (sEMG) to examine acute changes in muscle coordination following digital sports dance sessions accompanied by groove music. We further investigate how these changes relate to TGMD-3 scores and cognitive performance, aiming to provide practical insights for future physical activity interventions.

We hypothesize that the Groove Music + Sports Dance (GODA) intervention will lead to greater improvements in gross motor skills (TGMD-3), executive function (BRIEF and Stroop Test), and muscle coordination (sEMG coherence) compared to both Conventional Music + Sports Dance (CODA) and a non-musical Control Activity (CON). The proposed mechanisms include rhythmic anchoring, through which groove music provides a temporal framework that reduces motor planning demand, and emotional enhancement via music-induced dopamine release that supports sustained attention.

## 2. Methods

### 2.1. Participants

The required sample size was determined a priori using G*Power software (version 3.1.9.7). Based on an anticipated medium effect size (ES = 0.40) for the primary outcomes (changes in gross motor skills and executive function), an alpha level of 0.05, and a desired power of 0.80 for the planned repeated-measures analysis of variance, a minimum of 26 participants was estimated. To accommodate potential attrition and ensure sufficient statistical power for the more complex muscle coordination analyses, a total of 38 typically developing school-age children (aged 7 to 12 years) were recruited. Recruitment was conducted in collaboration with multiple primary schools, community centers, and youth activity centers in the target region. Participants were included if they met the following criteria: (1) age between 7 and 12 years; (2) absence of significant neuromuscular, skeletal, or cardiovascular disorders that would preclude safe participation in moderate-intensity physical activity like digital sports dance; (3) normal or corrected-to-normal vision and hearing sufficient for task engagement. Exclusion criteria were as follows: (1) severe visual, auditory, or other uncorrected sensory deficits significantly impairing participation; (2) diagnosed cognitive, learning, or neurodevelopmental disorders (e.g., autism spectrum disorder, attention deficit hyperactivity disorder—ADHD, and intellectual disability) potentially affecting the ability to follow instructions or perform tasks; (3) current participation in other structured interventions (e.g., intensive sports training, cognitive training programs) likely to influence gross motor development, executive function, or neuromuscular control patterns within the study period. Prior to enrollment, comprehensive written and verbal information detailing the study’s purpose, procedures, potential risks, and benefits was provided to the parents or legal guardians of all prospective participants. Written informed consent was obtained from all guardians, and verbal assent was obtained from all children, confirming their understanding and willingness to participate. The study protocol received full ethical review and approval from the Ethics Committee of Beijing Normal University (Approval No. BNU20241125) and strictly adhered to the ethical principles outlined in the Declaration of Helsinki.

### 2.2. Research Design

**Figure 1:**
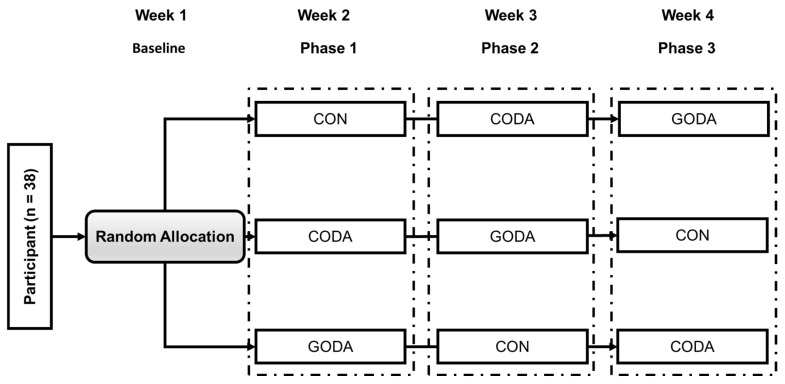
Flowchart of experimental allocation. Abbreviations: GODA: Groove Music + Sports Dance Condition; CODA: Conventional Music + Sports Dance Condition; CON: Control Condition.

This study employed a randomized repeated-measures design with counterbalanced condition order to evaluate the acute effects of three experimental conditions on children’s gross motor skills, executive function, and muscle coordination patterns. All 38 participants completed all three 45 min sessions in a randomized sequence determined by computer-generated Latin squares: (1) Groove Music + Sports Dance (GODA) using digital dance intervention with rhythmically accentuated music (prominent bass lines and syncopation), (2) Conventional Music + Sports Dance (CODA) featuring identical choreography but with non-groove background music matched in tempo, and (3) Control Activity (CON) involving matched-intensity brisk walking/non-rhythmic games devoid of music and dance elements. Each session was separated by a 5-day washout period and conducted in a standardized laboratory environment by certified instructors. Immediately before and after each session, participants underwent standardized assessments of gross motor skills, executive function, and muscle coordination. The counterbalanced order controlled for sequence effects while enabling within-subject comparisons across all conditions through pre/post measurements (Figure 1). The study was conducted over a 4-week period. Each 45 min session was separated by a standardized 5-day washout period to minimize carryover effects. Baseline assessments were conducted during the initial screening visit. Post-intervention testing was performed immediately (within 15 min) after each session under identical conditions. This design resulted in a total of seven testing timepoints per participant: one baseline assessment, and two pre- and post-test assessments for each of the three conditions (GODA, CODA, and CON). All assessments were completed within the 4-week timeframe. The standardized timing of assessments (always at the same time of day) was used to control for circadian variations in performance.

### 2.3. Digital Dance Intervention Protocol

The digital dance intervention for this study employed the “Intergalactic Energy Rescue Mission,” an immersive augmented reality (AR)-based dynamic gymnastics program, as its core platform across three experimental conditions that differed primarily in their musical components and synchronization requirements. (1) For the Groove Music + Sports Dance (GODA) condition, music selection followed a standardized procedure where candidate tracks scoring > 90 on a 1–127 scale from Janata’s music database were initially selected based on strong rhythmic properties and high engagement potential, and then evaluated by music experts for age-appropriateness (120–140 BPM tempo, 20–30% syncopation), with the lyrics removed using BrevAI software V 3.5 and final tracks requiring > 4.5 on Janata’s groove perception scale. (2) The Conventional Music + Sports Dance (CODA) condition used identical choreography with non-groove background music matched only in tempo (120–140 BPM) without significant syncopation or bass emphasis. (3) The Control (CON) condition involved matched-intensity brisk walking/non-rhythmic games without music. All dance conditions involved certified instructor supervision and were implemented via floor/wall-projected AR systems that translated six gross motor skills into immersive tasks (accelerated curved-path running, single-leg hopping, side-sliding, standing long jump, two-handed catching, and stationary ball kicking). Movement design was rigorously aligned with TGMD-3 indicators but differed in their synchronization demands: GODA movements were tightly coupled to musical rhythm with beat-synchronized AR feedback, CODA movements followed spatial accuracy with movement-triggered AR feedback, and CON activities had no rhythmic or technological synchronization elements.

**Figure 2:**
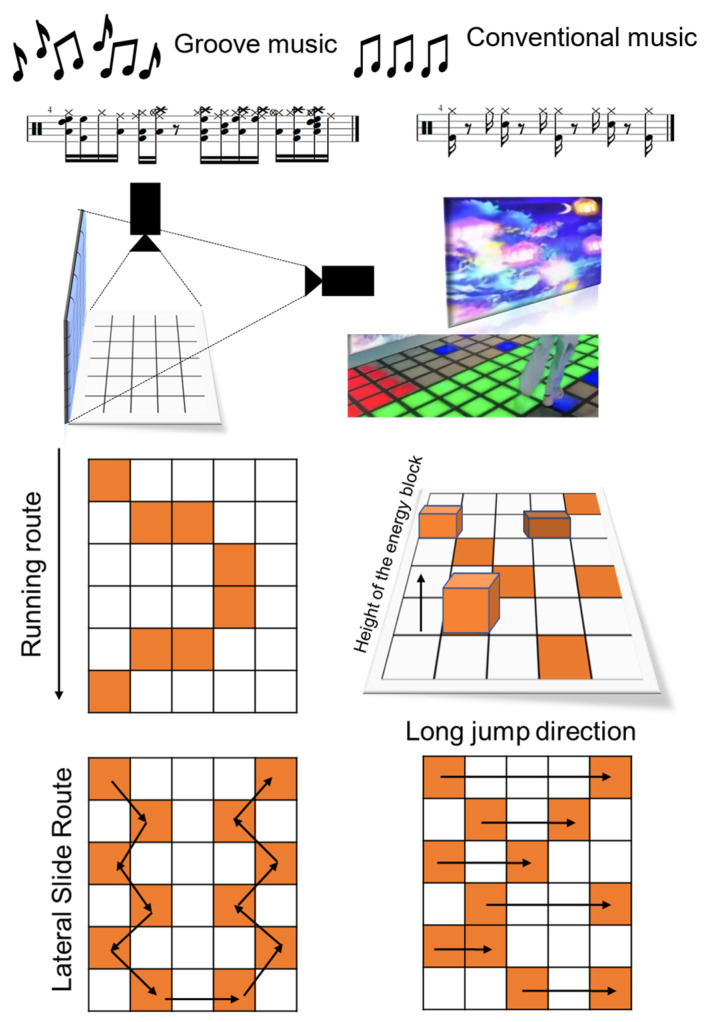
Schematic illustration of AR-integrated sports dance tasks. Movement trajectories are indicated by arrows, with tasks 5 and 6 implemented via wall-mounted projection systems.

Movement design specifically included the following: (1) accelerated curved-path running (chasing moving energy blocks), (2) single-leg hopping (touching variably suspended energy blocks), (3) side-sliding (sequentially collecting zigzag-arranged energy blocks), (4) standing long jump (cleaving ground fissures to land on energy block generation points), (5) two-handed catching (intercepting aerially descending energy blocks), and (6) stationary ball kicking (propelling “meteorite” energy blocks toward target trajectories) (Table 1). All tasks were executed in accordance with standardized procedures and under the supervision of certified instructors (see Figure 2).

### 2.4. Assessment Indices

#### 2.4.1. Gross Motor Development

##### TGMD-3 (Third Edition of the Test of Gross Motor Development)

The TGMD-3, a standardized tool, assessed children’s gross motor skills, focusing on locomotor and object-control skills. It comprised 13 items: 6 locomotor (e.g., running and hopping) and 7 object-control (e.g., throwing and catching). Each item had 3–5 criteria, scored as “1” for met criteria, tested twice, and totaled for item scores. Locomotor skills had a max score of 46; object-control, 54; combined total, 100. This score was converted to a standard score for evaluating gross motor development.

Before testing, children warmed up with light jogging and stretching to prevent injuries. A trained tester observed and scored each child’s performance using the TGMD-3 manual, ensuring accuracy and consistency.

#### 2.4.2. Executive Function

##### Behavior Rating Inventory of Executive Function (BRIEF)

The BRIEF, a standardized parent-reported questionnaire, assessed children’s executive function across five dimensions: emotional regulation, planning/organization, working memory, inhibitory control, and flexibility. Using a Likert scale (“never” to “always”), it quantified executive function behaviors.

##### Stroop Test

The Stroop Test evaluated inhibitory control via four parts: word naming, color naming, color interference, and word interference. Children responded to stimuli on a computer screen, with software recording reaction times and accuracy. The interference effect, calculated as the difference between interference task time and naming time, measured inhibitory control. Specifically, color interference was the time difference between Parts 1 and 3, and word interference was the difference between Parts 2 and 4.

#### 2.4.3. Muscle Coordination Assessment

**Figure 3:**
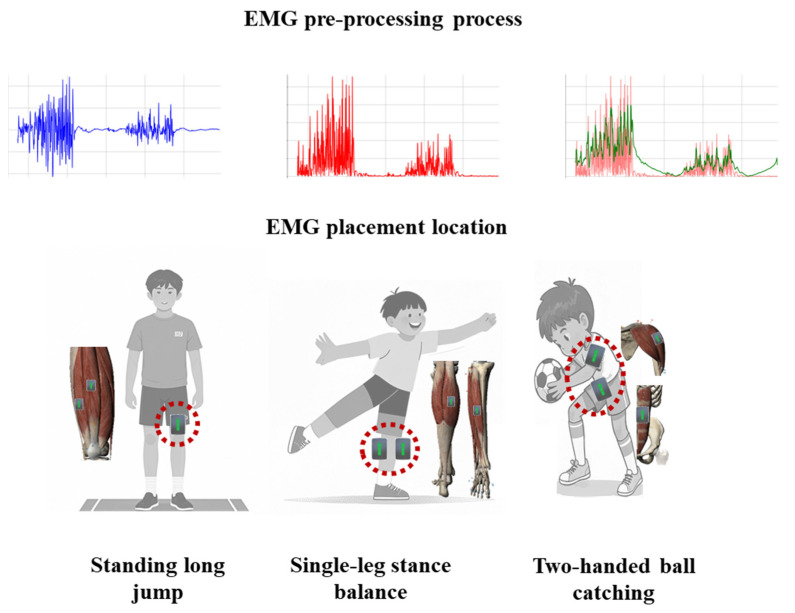
Schematic illustration of neuromuscular control tasks and electromyography (EMG) electrode placement. Note: Due to privacy regulations, cartoon representations were used instead of photographs of pediatric participants.

Muscle coordination was evaluated using wireless surface electromyography (sEMG; Delsys Trigno) during three TGMD-3-aligned tasks: standing long jump, single-leg stance balance, and two-handed ball catching, targeting lower-limb explosive power, ankle stability, and upper-limb coordination, respectively. Bipolar electrodes were placed parallel to muscle fibers (20 mm inter-electrode distance) over the quadriceps femoris (vastus lateralis (VL) and rectus femoris (RF)) for jumping, gastrocnemius (GAS)-tibialis anterior (TA) for balancing, and anterior deltoid (AD)-rectus abdominis (AB) for catching. The surface EMG locations are shown in Figure 3.

The primary metric of muscle coordination was intermuscular time–frequency coherence (TFC) between muscle pairs, calculated using Halliday’s unified framework. Raw sEMG signals were bandpass-filtered (20–450 Hz), full-wave-rectified, and smoothed (50 ms RMS window), followed by envelope extraction via a 10 ms moving average. Coherence was computed as follows:

$$Coherence(f)=\frac{{\left|S_{xy}(f)\right|}^{2}}{S_{xx}(f)\cdot S_{yy}(f)},$$

where *S_xy_*(*f*) is the cross-spectral density, and *S_xx_*(*f*), *S_yy_*(*f*) are auto-spectral densities. Significant coherence zones (threshold: >0.5) within α (8–15 Hz), β (15–30 Hz), and γ (30–50 Hz) bands were identified, and the area under the coherence curve (AZ) was quantified. Event markers (e.g., jump onset, ball impact) were synchronized with motion capture data. This protocol provides a multiband perspective on neuromuscular coordination, enabling mechanistic exploration of gross motor control.

In addition to intermuscular coherence, muscle co-activation, integral electromyography (IEMG), and median frequency (MDF) were also analyzed to provide a more comprehensive understanding of muscle coordination. Muscle co-activation was assessed within agonist–antagonist pairs by calculating the ratio of integrated EMG signals between the antagonist and agonist muscles over the defined task epochs. Lower muscle co-activation ratios indicate more efficient reciprocal activation. The total muscle activation level during each task was quantified by integrating the full-wave-rectified and smoothed sEMG signal over the defined task epoch. The median frequency of the power spectrum of the raw sEMG signal was calculated over the defined task epoch using Fast Fourier Transform (FFT). A higher median frequency generally indicates faster motor unit recruitment or less fatigue.

The neuromuscular control tasks in this study—standing long jump, single-leg stance balance, and two-handed ball catching—were systematically selected based on the following criteria: alignment with TGMD-3 Assessment Items, as the chosen tasks directly corresponded to core motor skills in TGMD-3 (e.g., standing long jump for “locomotor skills” and ball catching for “object-control skills”), ensuring consistency between motor performance evaluation and intervention content; typical demands of muscle coordination, with the standing long jump assessing lower-limb explosive power (co-activation of vastus lateralis, VL, and rectus femoris, RF) and landing stability, the single-leg stance balance evaluating ankle stability (antagonistic coordination among gastrocnemius, GAS, and tibialis anterior, TA), and the two-handed ball catching reflecting upper-limb–trunk coordination (temporal synchronization of anterior deltoid, AD, and rectus abdominis, AB); and safety for child execution, as task complexity was adapted to the ability level of children aged 7–12 years, excluding high-risk movements. This selection framework was supported by prior research, where similar tasks have been used in studies of children’s motor development [4,5], and β-/γ-band coherence analysis has proven effective in quantifying neuromuscular coordination [18,19].

### 2.5. Statistical Analysis

Outliers were identified via boxplot inspection and winsorized at the 90th percentile. The Shapiro–Wilk test confirmed non-normality in the data distribution, justifying the use of non-parametric analyses. For each assessment timepoint, three types of difference scores were calculated using data from all six valid trials per participant. Friedman tests were employed to assess differences in change scores (post-pre) across the three experimental conditions (GODA, CODA, and CON) for each outcome variable (gross motor skills, executive function, muscle coordination). For outcomes yielding significant Friedman test results, post hoc pairwise comparisons were conducted using Wilcoxon signed-rank tests with Holm–Bonferroni correction for multiple comparisons. The tables show raw scores for reference, but the change scores (i.e., the difference between pre- and post-test) were used in the statistical comparisons. To examine relationships between key outcome variables, Spearman rank-order correlation coefficients were computed. All analyses were performed using SPSS Statistics version 26.0, with the significance level (α) set at 0.05.

## 3. Results

### 3.1. Gross Motor Development

Table 2 demonstrates comparative TGMD scores across conditions. Locomotor subscale: Post-intervention condition differences were statistically significant (χ^2^(2) = 12.34, *p* < 0.001). Both the GODA (Groove) and CODA (Traditional) conditions showed significant improvements compared to the CON (Silent) condition, with the GODA condition outperforming the CODA condition (Z = −2.14, *p* = 0.03). Ball skill subscale: Post-intervention condition differences were highly significant (χ^2^(2) = 15.67, *p* < 0.001). The GODA and CODA conditions exhibited superior performance relative to the CON condition, with the GODA condition demonstrating significantly greater improvement than the CODA condition (Z = −2.34, *p* = 0.02).

### 3.2. Executive Function

Table 3 presents BRIEF score comparisons. Inhibition and shifting dimensions showed significant post-intervention condition differences (*p* < 0.05). Both GODA and CODA conditions exhibited reduced scores compared to the CON condition, though no significant differences were observed between the two intervention conditions. Working memory demonstrated highly significant post-intervention condition differences (χ^2^(2) = 18.90, *p* < 0.001). The GODA condition achieved significantly lower scores than both the CODA (Z = −2.56, *p* = 0.01) and CON conditions. Planning showed similar trends (χ^2^(2) = 17.23, *p* < 0.001), with the GODA condition outperforming the CODA condition (Z = −2.10, *p* = 0.03). Organization revealed significant post-intervention differences (χ^2^(2) = 10.23, *p* = 0.02), with the CODA condition showing reduced scores compared to the CON condition (Z = −2.05, *p* = 0.04).

### 3.3. Stroop Test

Table 4 summarizes Stroop Test outcomes. Color interference: No significant post-intervention condition differences were observed (χ^2^(2) = 2.34, *p* = 0.31). Word interference: The GODA condition demonstrated significantly reduced reaction times compared to the CON condition (Z = −2.01, *p* = 0.04).

### 3.4. Muscle Coordination

**Figure 4:**
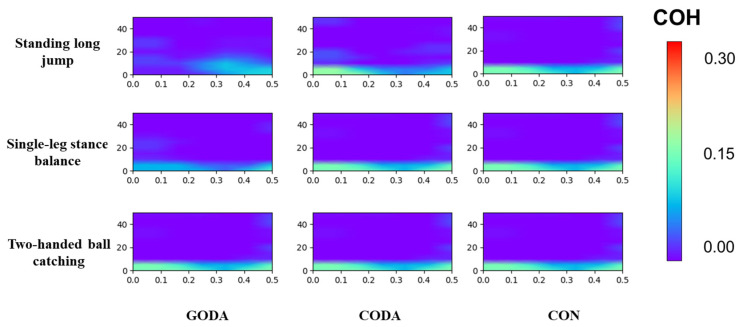
Intermuscular coherence comparisons across conditions. COH values represent the coherence area. Non-significant coherence values (*p* ≥ 0.05) are masked in dark blue.

**Figure 5:**
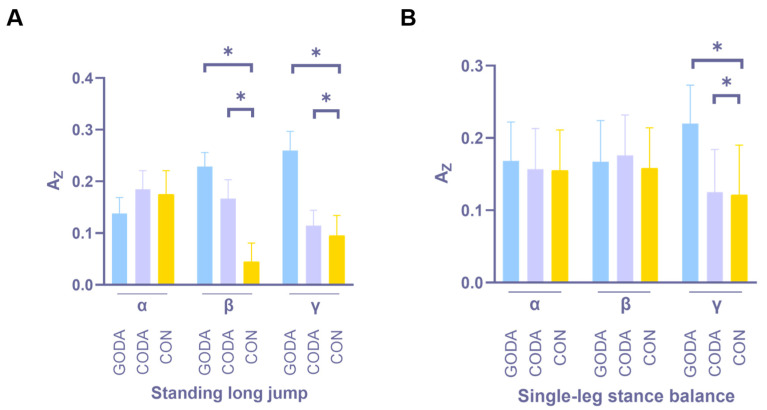
Tasks showing significant between-condition differences in frequency band coherence areas. Only post-intervention comparisons are displayed for brevity. * indicates a significant difference between the two groups. (**A**) is Standing long jump, (**B**) is Single-leg balance.

Figure 4 and Figure 5 illustrate muscle coordination task comparisons using intermuscular coherence (COH) across frequency bands. Standing long jump: GODA exhibited greater β- and γ-band COH areas than both CODA (*p* = 0.02 for both bands) and CON (*p* = 0.02 and *p* < 0.001, respectively). Single-leg balance: GODA showed increased γ-band COH areas compared to CODA (*p* = 0.02) and CON (*p* < 0.001).

**Figure 6:**
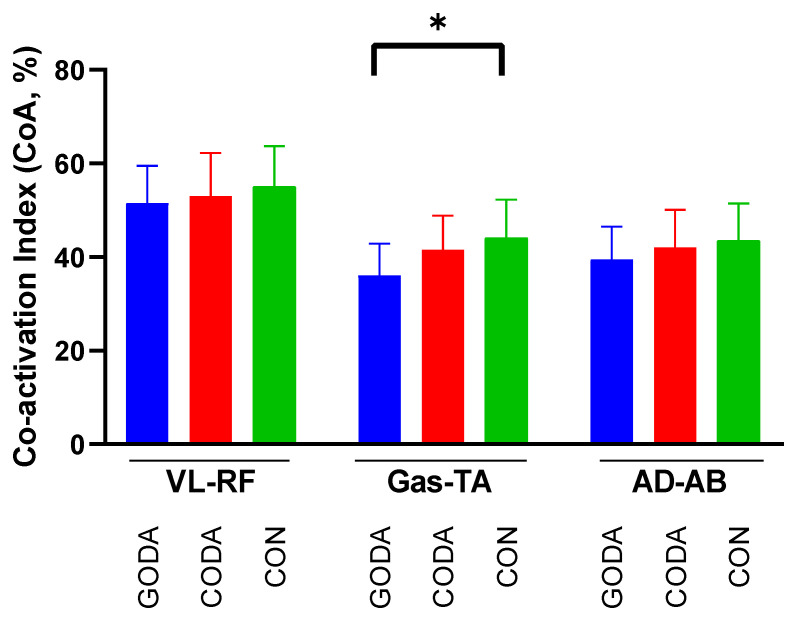
Muscle co-activation index (CoA, %) comparison across conditions. * indicates a significant difference between the two groups.

Friedman tests revealed limited significant differences in muscle co-activation indices across conditions (Table 5 and Figure 6). While overall comparisons showed marginal effects (*p* = 0.06–0.08), post hoc analysis identified a single consistent pattern:

Balance task: GODA demonstrated significantly reduced gastrocnemius-tibialis anterior (Gas-TA) co-activation compared to CON (*p* = 0.03).

Integral EMG (iEMG): Analysis of muscle activation levels showed no significant condition differences across all monitored muscles (Table 6).

Median Frequency (MF): Neuromuscular recruitment patterns were homogeneous across conditions (Table 7). The only notable trend was as follows: 6% higher tibialis anterior (TA) median frequency during balance in GODA vs. CON (*p* = 0.11).

**Figure 7:**
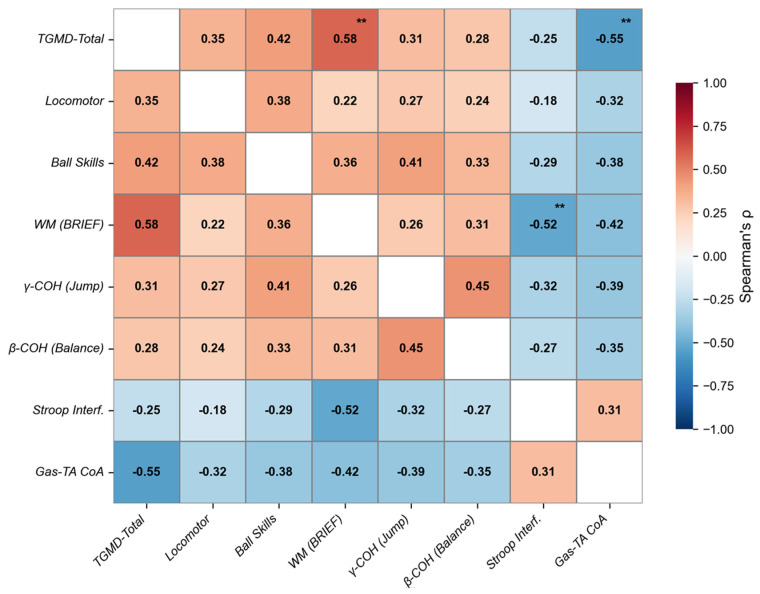
Correlation network of key outcome variables following GODA intervention. Abbreviations: WM = working memory; γ-COH = gamma-band coherence; β-COH = beta-band coherence; Gas-TA CoA = gastrocnemius-tibialis anterior co-activation index. ** indicates a significant difference between the two groups.

Spearman’s correlation analysis revealed three statistically significant relationships following the GODA intervention (Figure 7). The TGMD total score demonstrated a strong positive correlation with working memory (r = 0.58, *p* < 0.01). Concurrently, TGMD scores showed a significant negative correlation with gastrocnemius-tibialis anterior co-activation (r = −0.55, *p* < 0.01). Furthermore, working memory was inversely associated with Stroop interference (r = −0.52, *p* < 0.01).

## 4. Discussion

In this study, we systematically compared the effects of GODA, CODA, and CON interventions on the gross motor development, executive function, and muscle coordination of schoolchildren. The results demonstrated that while the GODA condition showed advantages in specific aspects of locomotor skills compared to CODA, both GODA and CODA interventions significantly improved ball skills relative to CON. These findings support the theoretical framework of “auditory–motor coupling” [20], particularly highlighting how groove music’s rhythmic structure may facilitate specific movement patterns through enhanced sensorimotor integration. The modest but consistent differences suggest potential clinical relevance for targeted motor skill interventions, where even small improvements could benefit children with movement difficulties [21].

The GODA intervention showed selective advantages in executive function, particularly in working memory, compared to CODA. Interestingly, CODA also demonstrated benefits in organization relative to CON, suggesting that different music characteristics may influence distinct cognitive domains. These results align with previous findings that rhythmic structure can affect cognitive processing [22], though the specific mechanisms require further investigation. The observed β- and γ-band coherence enhancements in GODA may reflect improved cortico-subcortical communication, particularly in fronto-parietal networks involved in working memory [23]. The increased coherence likely indicates more efficient neural synchronization between motor planning and execution areas, potentially mediated by rhythmic entrainment of cerebellar-thalamo-cortical loops [24].

In the Stroop Test, the GODA condition showed reduced reaction times specifically in the word interference task. This task-specific improvement parallels findings that rhythmic auditory stimuli may preferentially influence verbal processing networks [25]. The absence of effects in color interference suggests that these benefits may be domain-limited, consistent with other studies showing selective cognitive effects of musical interventions [26]. The γ-band coherence changes observed during motor tasks may extend to language-related cortical areas, facilitating faster lexical access during verbal tasks.

Muscle coordination analysis revealed that GODA enhanced neuromuscular synchronization during dynamic tasks compared to both CODA and CON, with CODA showing intermediate values (Figure 5). The increased β-band coherence suggests improved motor planning and preparation at cortical levels, while γ-band enhancements may reflect more precise timing of muscle activation patterns through spinal and brainstem pathways [27]. These results provide concrete evidence for music’s role in movement coordination [28], extending previous work on rhythm and motor control [29]. The gradient of effects across conditions suggests a dose–response relationship between rhythmic complexity and neuromuscular synchronization, with groove music providing optimal temporal cues for motor unit recruitment.

When compared to similar interventions [30], our findings demonstrate that groove music offers particular advantages for specific motor and cognitive functions, while conventional music retains some benefits over non-musical activities. This pattern has been observed in other movement-based interventions incorporating rhythmic components [28]. However, as these results represent acute effects, future longitudinal studies should investigate whether these benefits persist with continued training and whether they lead to lasting neuroplastic changes. Particularly important would be examining the retention of neuromuscular synchronization patterns and their transfer to untrained motor tasks.

The integration of these results supports the potential value of music-based interventions for children’s development. The condition-specific effects we observed, particularly GODA’s advantages in locomotor skills and working memory, along with CODA’s benefits in organization and intermediate neuromuscular effects, suggest that different music characteristics may be strategically employed depending on intervention goals. These findings contribute to a growing body of research demonstrating the benefits of rhythm-enhanced activities for motor and cognitive development [29], while also highlighting the need for further investigation into the specific mechanisms underlying these effects. Future research should employ longer intervention periods with follow-up assessments to determine the sustainability of these effects and their potential clinical applications for children with developmental coordination disorders or attention deficits.

### Limitations

While this study provides valuable insights into the acute effects of rhythm-enhanced dance interventions, several limitations should be noted. First, the participant sample was relatively homogeneous, consisting of typically developing children from a specific geographic region. Future research should include more diverse populations to enhance the generalizability of the findings. Second, individual differences in baseline motor coordination, musical experience, or cognitive abilities may influence responsiveness to the intervention. Exploring these factors in future studies could help identify subgroups that benefit most from groove-based training and inform personalized intervention approaches.

## 5. Conclusions

In conclusion, the GODA intervention, with its unique combination of groove music and sports dance, significantly enhances gross motor skills, executive function, and neuromuscular coordination in school-aged children. These findings advocate for the inclusion of rhythmically accentuated music in physical education and cognitive intervention programs, emphasizing its role in optimizing both motor and cognitive outcomes. Future research should explore the long-term effects of such interventions and their applicability to diverse populations.

## Figures and Tables

**Table 1:** Design of sports dance intervention program.

Training Objectives	AR Tasks
1. Running	The energy blocks move along a curved path, and one needs to accelerate to catch up.
2. Single-leg Hopping	The suspended energy blocks are arranged in a scattered manner with different heights. Touch them by hopping on one leg.
3. Side Sliding	The energy blocks are arranged in a zigzag shape. Collect them continuously by side sliding.
4. Standing Long Jump	One needs to jump over the cracks on the ground, and the landing point is where the energy blocks are generated.
5. Two-handed Ball Catching	The energy blocks fall from the air. Catch them by making a ball-catching gesture with both hands.
6. Kicking a Fixed Ball	The energy blocks turn into “meteorites”. Swing the leg and kick towards the target track.

**Table 2:** Comparison of TGMD scores across conditions.

	GODA	CODA	CON	*p*-Value
Locomotor	11.5 (2.50)	10.5 (2.50)	9.0 (2.00)	<0.001
**Post hoc**	*p* = 0.03 (GODA vs. CODA)	*p* = 0.01 (GODA vs. CON)	*p* = 0.02 (CODA vs. CON)	
Ball skills	17.0 (2.50)	16.0 (2.75)	14.0 (2.25)	<0.001
**Post hoc**	*p* = 0.02 (GODA vs. CODA)	*p* = 0.02 (GODA vs. CON)	*p* = 0.04 (CODA vs. CON)	

GODA: Groove Music + Sports Dance Condition; CODA: Conventional Music + Sports Dance Condition; CON: Control Condition.

**Table 3:** Comparison of BRIEF scores across conditions.

	GODA	CODA	CON	*p*-Value
**Inhibition**	14.00 (6.25)	13.00 (7.25)	15.00 (6.50)	0.04
**Post hoc**	*p* = 0.22 (GODA vs. CODA)	*p* = 0.04 (GODA vs. CON)	*p* = 0.03 (CODA vs. CON)	-
**Shifting**	10.50 (4.25)	10.50 (3.75)	11.00 (2.50)	0.03
**Post hoc**	*p* = 0.57 (GODA vs. CODA)	*p* = 0.03 (GODA vs. CON)	*p* = 0.04 (CODA vs. CON)	-
**Emotional control**	15.50 (6.50)	14.50 (6.75)	16.00 (5.50)	0.05
**Initiation**	12.50 (4.50)	11.50 (4.25)	13.00 (3.00)	0.06
**Working memory**	18.00 (6.50)	19.25 (5.50)	18.50 (6.25)	<0.001
**Post hoc**	*p* = 0.01 (GODA vs. CODA)	*p* = 0.02 (GODA vs. CON)	*p* = 0.06 (CODA vs. CON)	-
**Planning**	20.50 (7.25)	19.25 (6.50)	19.50 (6.50)	<0.001
**Post hoc**	*p* = 0.03 (GODA vs. CODA)	*p* = 0.37 (GODA vs. CON)	*p* = 0.54 (CODA vs. CON)	-
**Organization**	8.50 (4.25)	7.50 (5.00)	9.00 (3.50)	0.02
**Post hoc**	*p* = 0.21 (GODA vs. CODA)	*p* = 0.34 (GODA vs. CON)	*p* = 0.04 (CODA vs. CON)	-
**Monitoring**	11.50 (5.50)	12.50 (5.00)	11.00 (4.50)	0.07

**Table 4:** Comparison of Stroop Test scores across conditions.

	GODA	CODA	CON	*p*-Value
Color interference (s)	5.50 (4.50)	4.50 (3.75)	4.50 (3.00)	0.31
Word interference (s)	19.00 (10.50)	19.50 (11.00)	20.00 (10.50)	0.04
Post hoc	-	-	*p* = 0.04 (GODA vs. CON)	-

**Table 5:** Muscle co-activation index (CoA, %) comparison.

Task	Muscle Pair	GODA	CODA	CON	*p*-Value	Post Hoc
Jump (Landing)	**VL-RF**	51.5 (8.0)	53.0 (9.2)	55.0 (8.7)	0.21	-
Balance	**Gas-TA**	36.0 (6.8)	41.5 (7.3)	44.0 (8.2)	0.03	*p* = 0.03 (GODA vs. CON)
Catch (Prep)	**AD-AB**	39.5 (7.0)	42.0 (8.1)	43.5 (7.9)	0.17	-

**Table 6:** Integral EMG comparison (iEMG, μV·s).

Task	Muscle	GODA	CODA	CON	*p*-Value
Jump	Vastus Lateralis	1050 (160)	980 (150)	970 (145)	0.12
Balance	Gastrocnemius	470 (80)	505 (85)	510 (90)	0.18
Catch	Rectus Abdominis	600 (100)	580 (95)	570 (90)	0.25
Jump	Rectus Femoris	390 (65)	410 (70)	420 (72)	0.32
Balance	Tibialis Anterior	330 (55)	350 (58)	355 (60)	0.21
Catch	Anterior Deltoid	290 (48)	300 (50)	305 (52)	0.35

**Table 7:** Median frequency comparison (MF, Hz).

Task	Muscle	GODA	CODA	CON	*p*-Value
Balance	Tibialis Anterior	82.0 (11.0)	79.5 (10.5)	77.5 (10.0)	0.11
Catch	Anterior Deltoid	88.0 (12.8)	86.5 (12.0)	85.5 (11.5)	0.23
Jump	Vastus Lateralis	75.0 (10.5)	74.5 (10.2)	73.5 (9.8)	0.42
Balance	Gastrocnemius	69.0 (9.5)	68.5 (9.2)	67.5 (8.8)	0.37
Catch	Rectus Abdominis	66.0 (8.8)	65.5 (8.5)	64.5 (8.0)	0.29
Jump	Rectus Femoris	63.0 (8.0)	62.5 (7.8)	61.5 (7.2)	0.33

## Data Availability

All data in this paper are presented in the manuscript.
